# *In silico* analysis of design of experiment methods for metabolic pathway optimization

**DOI:** 10.1016/j.csbj.2024.04.062

**Published:** 2024-05-03

**Authors:** Sara Moreno-Paz, Joep Schmitz, Maria Suarez-Diez

**Affiliations:** a Laboratory of Systems and Synthetic Biology, Wageningen University & Research, 6708WE Wageningen, the Netherlands; b Department of Science and Research - dsm-firmenich, Science & Research, 2600 MA Delft, the Netherlands

**Keywords:** Design of experiments, Pathway, Cell factory

## Abstract

Microbial cell factories allow the production of chemicals presenting an alternative to traditional fossil fuel-dependent production. However, finding the optimal expression of production pathway genes is crucial for the development of efficient production strains. Unlike sequential experimentation, combinatorial optimization captures the relationships between pathway genes and production, albeit at the cost of conducting multiple experiments. Fractional factorial designs followed by linear modeling and statistical analysis reduce the experimental workload while maximizing the information gained during experimentation. Although tools to perform and analyze these designs are available, guidelines for selecting appropriate factorial designs for pathway optimization are missing. In this study, we leverage a kinetic model of a seven-genes pathway to simulate the performance of a full factorial strain library. We compare this approach to resolution V, IV, III, and Plackett Burman (PB) designs. Additionally, we evaluate the performance of these designs as training sets for a random forest algorithm aimed at identifying best-producing strains. Evaluating the robustness of these designs to noise and missing data, traits inherent to biological datasets, we find that while resolution V designs capture most information present in full factorial data, they necessitate the construction of a large number of strains. On the other hand, resolution III and PB designs fall short in identifying optimal strains and miss relevant information. Besides, given the small number of experiments required for the optimization of a pathway with seven genes, linear models outperform random forest. Consequently, we propose the use of resolution IV designs followed by linear modeling in Design-Build-Test-Learn (DBTL) cycles targeting the screening of multiple factors. These designs enable the identification of optimal strains and provide valuable guidance for subsequent optimization cycles.

## Graphical abstract

**Figure:**
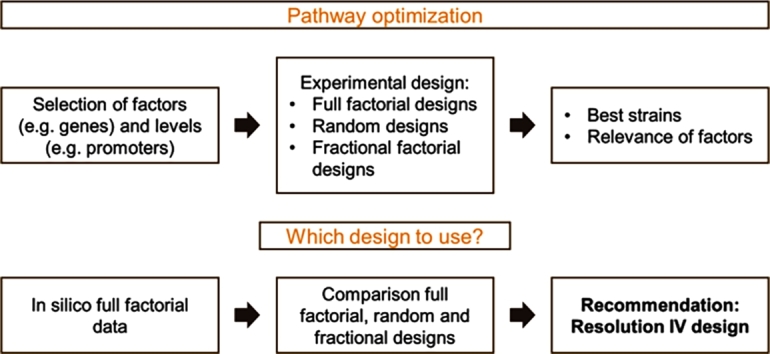

## 1 Introduction

Industrial biotechnology uses microbial cell factories for sustainable chemical production often through the expression of heterologous pathways in a microbial host. However, a common challenge when introducing a pathway in a microorganism is to find the optimal expression level of each of the introduced genes [1], [2], [3]. This question can be answered using sequential or combinatorial experimentation, depending on whether the expression of the genes is optimized individually or simultaneously. When combinatorial optimization is used, the likelihood of finding the optimal expression levels increases [4], [5]. For example, if the abundance of protein A is limiting the pathway, the expression of other pathway genes will not affect production as long as the expression of A is low. However, when the expression of gene A increases, changes in the expression of other pathways genes will likely affect production. Combinatorial pathway optimization captures these interactions between the pathway genes and can better guide the pathway optimization process.

Combinatorial optimization requires the construction of numerous strains. When optimizing a pathway with three genes and two expression levels, constructing eight (2^3^) strains is needed to test all the combinations of genes and levels (full factorial design). The number of strains to construct increases exponentially with the number of genes to optimize. If the number of genes increases to seven, the number of strains increases to 128 (2^7^). Moreover, the number of strains to build increases even faster when more than two expression levels are tested: 27 (3^3^) strains for three genes with three expression levels and 2187 (3^7^) for seven genes with three expression levels. Even with efficient and automated strain construction and characterization pipelines, reducing the number of strains to build and test while maintaining the ability to discern the relative importance of the pathway genes and the presence of interactions is desired [4], [5].

Statistical design of experiments (DoE) is a technique to minimize the experimental effort while maximizing the information gained over the studied system [6]. This method can be easily incorporated in the Design-Build-Test-Learn (DBTL) cycles commonly used in industrial biotechnology [1], [2]. In the first cycle, factors, levels, and the desired response are defined. For example, a factor is each of the genes whose expression will be optimized, and a level is each of the expression strengths that will be tested. In turn, the response might be the final concentration of the target metabolite. The number of experiments to perform is equivalent to the number of strains to build and test. This number increases faster with the number of levels than the number of factors. Therefore, DoE often starts studying two levels per factor. In this way, more factors can be screened, important factors are identified, and the fine-tuning of factor levels is targeted only to the relevant factors in subsequent DBTL cycles. The information gained during experimentation is stored in a polynomial model. The model coefficients are fit so the response is a function of each of the factors and their interactions. In this model, the main effects are the coefficients that explain how the response is affected by changing each individual factor (gene). Similarly, two-factor interactions are coefficients that explain how the response changes simultaneously considering the levels of two factors (genes). Then, an analysis of variance (ANOVA) is used to quantify whether each model parameter significantly influences the response [6], [5]. The extent to which a significant factor influences the response is determined by the absolute value of its main effect. The sign of the coefficient indicates whether the factor has a positive or negative impact on the response. When two or multiple factor interactions are significant, the effect of a factor is also influenced by the interaction coefficients.

**Fig. 1:**
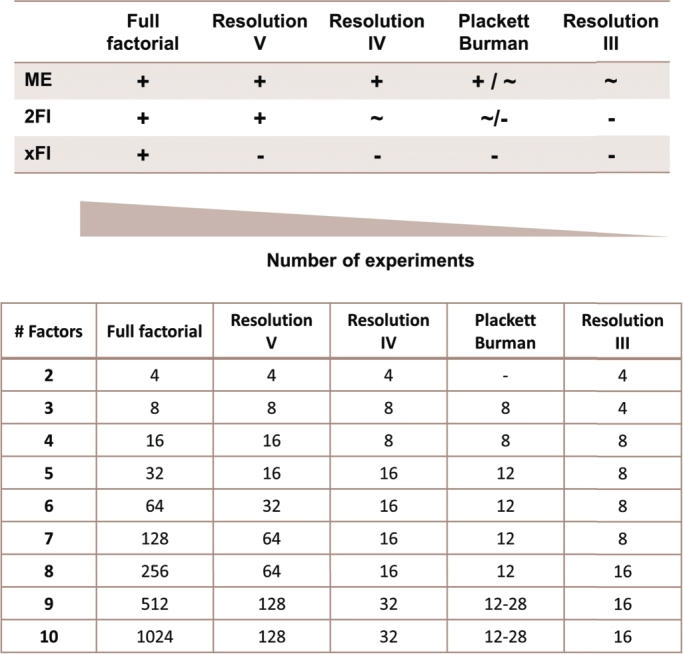
Ability of different fractional factorial designs to estimate main effects (ME), two-factor interactions (2FI), and higher-order interactions (xFI). + indicates the estimation of a coefficient clear of confounding, ∼ indicates a confounded estimation, and - indicates the inability to estimate this type of coefficient. In Plackett Burman designs confounded coefficients are partially correlated with each other, which allows the estimation of some of the interactions.

Factorial designs are used to efficiently sample the design space determined by the factors and levels and are useful for screening in initial DBTL cycles. Different factorial designs exist depending on the number of experiments to perform and the aliasing structure. Aliasing or confounding referees to model coefficients that are indistinguishable from each other. A design with a higher resolution requires the execution of more experiments and results in the confounding of only high-order interactions [6]. For instance, resolution V designs allow the clear identification of main effects and two-factor interactions. However, they confound three-order interactions among each other. This means that, although some three-factor interaction coefficients can be estimated, they cannot be assigned to a specific combination of factors. Similarly, resolution IV designs confound two-factor interactions among each other. Therefore, they can be used to assess whether these interactions are important but they cannot identify the specific interactions that influence the response. Resolution III designs confound main effects with two-factor interactions. Therefore, models including main effects can be created, but the estimated coefficients represent the mixed effect of the single factor and the confounded interactions. If interactions are not significant, low-resolution designs efficiently reduce the number of experiments. However, they may result in incorrect determination of main effects when interactions affect the response. Plackett Burman (PB) designs are a special type of resolution III designs in which two-factor interactions are partially confounded with main effects allowing the estimation of some interactions. A summary of DoE designs is presented in Fig. 1 [6].

Factorial designs can easily be generated and analyzed using web servers such as DATAtab, licensed programs (Minitab [7], JMP [8]) and open-source packages like FrF2 in R [9]. Moreover, these designs have been used for the optimization of expression of pathways genes [10], [2]. However, clear recommendations of the type of designs to use for pathway optimization are still missing, which hinders their widespread application [11].

*In silico* studies represent biological systems using mathematical models. This allows the simulation of multiple constructs and the evaluation of computational design tools [12], [13], [14]. These studies enable the characterization of the robustness of different design approaches to realistic biological scenarios where noise is present and problems during strain construction can lead to the inability to build some of the desired strains. The best strategies found by an *in silico* evaluation can then be applied in *in vivo* studies, in which the experimental throughput is considerably lower.

**Fig. 2:**
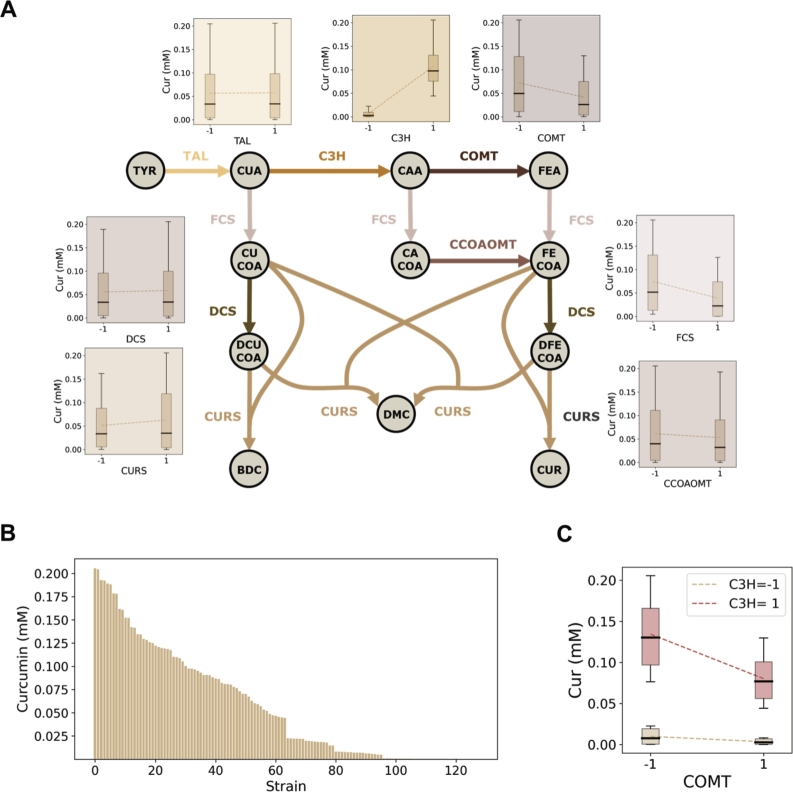
**A.** Curcuminoid pathway. Boxplots show the curcumin (Cur) production data distribution of strains containing low (-1) or high (1) enzyme concentration obtained with the kinetic model [15]. **B.** Kinetic model simulations of curcumin production of the 128 strains forming the full factorial design space. **C.** Production data distribution of strains containing low (-1) or high (1) concentration of COMT given low (-1) or high (1) concentration of C3H. Metabolite abbreviations: TYR, tyrosine; CUA, p-coumaric acid; CAA, caffeic acid; FEA, ferulic acid; CUCOA, coumaroyl-CoA; CACOA, caffeoyl-CoA; FECOA, feruloyl-CoA; DCUCOA, diketide coumaroyl-CoA; DFECOA, diketide feruloyl-CoA; BDC, bisdemethoxycurcumin; DMC, demethoxycurcumin; CUR, curcumin. Enzyme abbreviations: TAL, tyrosine ammonia lyase; C3H, coumarate-3-hydroxylase; COMT, caffeic acid O-methyl transferase; FCS, feruloyl/coumaroyl-CoA synthase; CCOAOMT, caffeoyl-CoA O-methyl transferase; DCS, diketide-CoA synthase; CURS, curcumin synthase.

Here, we use a mathematical kinetic model of the curcumin pathway (Fig. 2A) to simulate *in silico* a full factorial library of strains. This library consists of all the combinations of seven enzymes (factors) at two different concentrations (levels) [15]. This pathway is characterized by the presence of promiscuous enzymes that catalyze multiple reactions and the possibility to produce three different metabolites. Therefore, the effect of modifying the abundance of an enzyme on production is highly dependent on metabolite concentrations. Concentrations are, in turn, affected by the concentration of other pathway enzymes. The use of a kinetic pathway model enabled the identification of the best concentration levels of each enzyme, as well as the estimation of the real coefficients of the polynomial model. Considering this information, we tested the capacity of different factorial designs to find the best strains in the library space. We also tested their capacity to determine the coefficients of the model which could later be used to guide the expansion of the design space. With this we aim to aid metabolic engineers on their experimental designs to efficiently allocate time and resources.

## 2 Material and methods

### 2.1 Pathway simulation and noise

A kinetic model of the curcuminoid pathway was obtained from [15]. This model uses Michaelis-Menten kinetics expression rate laws for all reactions except C3H. For this reaction a mass-action rate law is used. Enzymes catalyzing multiple reactions (FCS, DCS, and CURS) contain additional substrate competition terms in their rate laws to account for their promiscuity (Fig. 2A). The model was simulated using the AMICI library [16] and the CVODES ODE solver [17]. Each of the seven enzymes in the pathway was considered a factor with the default enzyme concentration as *low* level and five times the default concentration as *high* level. The parameter corresponding to enzyme concentration for each reaction in the pathway was altered to simulate the 128 (2^7^) strains constituting the full factorial library. *In silico* triplicates for each strain were obtained adding 5% or 20% of Gaussian noise. The simulated full factorial data is available in Gitlab.

### 2.2 Simulation of DoE designs

Resolution V, IV, and III designs were generated using the FrF2 function from the FrF2 R package given the number of factors and the desired resolution [9]. Placket-Burman (PB) designs were generated with the pb function from the same package indicating the desired number of factors and experiments. For each design, columns were permuted to account for the effect of randomly assigning factors (enzymes) to the design columns. From the full factorial design data, experiments were selected according to the design and used to train a linear model by ordinary least squares regression using the R lm function. The linear model had the form:

(1) $$y=\beta_{0}+\sum_{i=1}^{i=n}ME_{i}\cdot F_{i}+\sum_{i=1}^{i=n}\sum_{j=1}^{j=n}2FI_{i:j}\cdot F_{i}\cdot F_{j},$$

where *y* represents the curcumin concentration obtained by the kinetic pathway model; $\beta_{0}$ represents the y-intercept, $ME_{i}$ refers to the main effect of factor *i* ($F_{i}$) and $2FI_{i:j}$ refers to the two-factor interaction between factor *i* and *j*. The total number of factors is indicated by *n*.

For resolution V and IV designs, additional linear models were trained assuming the inability to construct some of the proposed strains by randomly removing rows of the design matrix. The effect of excluding 1, 2, 5, and 10 rows or 1, 2, 3, and 5 rows for the resolution V and IV designs respectively was evaluated in 100 random permutations of the design columns.

To compare DoE designs with random sampling strategies, experiments from the full factorial design were randomly sampled using the sample function in R. The number of samples was equal to the number of strains selected by each of the designs and the sampling process was repeated as many times as the performed permutations.

For each permutation of the design or random sample, the R summary function was used to obtain the ANOVA table which provides the estimated coefficients of the linear model (MEs and 2FIs) and their associated p-values. These p-values and coefficients were compared to those obtained by training the linear model with the full factorial design (“ground truth”).

Linear models were evaluated on their ability to predict curcumin production of the full factorial library of strains. Main effects of models trained with fractional factorial designs or random samples were employed to calculate the curcumin concentration of all the strains in the full factorial library. Predictions of linear models were compared to the predictions of the kinetic model (“ground truth”) using the coefficient of determination (R^2^). The r2_score function from sklearn was used.

### 2.3 Prediction of optimal strains within the design space

Linear models trained with data derived from experiments selected by permutations of DoE designs or random sampling were used to predict strains with the highest curcumin production. Strains were predicted as optimal producers according to the linear models when the levels of the enzymes with a significant main effect agreed with the sign of the estimated coefficients in the linear model. For enzymes with insignificant main effects, strains containing any of the concentration levels were considered as optimal candidates. The frequency in which each strain was selected as optimal in each permutation of the design or set of random samples was computed and compared to the actual production according to the kinetic pathway model.

### 2.4 DoE and machine learning

The suitability of experiments designed using DoE to train machine learning (ML) models was assessed with random forest as an example using the scikit-learn Python library. Models were trained using 10-fold cross-validation and model performance was assessed based on the coefficient of determination (R^2^). Trained models were used to predict the production of the full factorial design space and the frequency of each strain as part of the two best predicted strains was computed. Additionally, random forest models were trained with all the data from the designs (without cross-validation). This approach was equivalent to the training strategy used for linear models. For 100 random permutations of the resolution IV design, the effect of hyper-parameter tuning on model performance was evaluated. A grid search using leave one out cross-validation was employed to optimize the number of estimators (100, 200, 200), the maximum depth of the trees (none, 10, 20), the minimum number of samples required to split an internal node (2, 5, 10) and the minimum number of samples required to be at a leaf node (1, 2, 4).

## 3 Results

### 3.1 Simulation of the full factorial library and factorial designs

The curcumin pathway contains seven enzymes from which FCS, DCS, and CURS are promiscuous and able to catalyze multiple reactions (Fig. 2A). Moreover, in this pathway demethoxycurcumin, bisdemethoxycurcumin, and curcumin can be produced. Therefore, optimizing curcumin production requires fine-tuning the concentration of the pathway enzymes. A full factorial library for this pathway considering two concentration levels per enzyme requires the simulation of 128 strains. This library contains all possible combinations between factor levels, resulting in curcumin production ranging from 10^−4^ to 0.2 mM. This library represents the “ground truth” for the system (Fig. 2B). In factorial designs, the effect of a factor is estimated considering replicate experiments and experiments where the given factor is constant regardless of other factor levels. For each enzyme, Fig. 2A shows the distribution of curcumin production by strains containing low (-1) or high (1) enzyme concentrations. Changing the concentration of C3H, COMT, and FCS has the highest impact on curcumin production, followed by changes in CCOAOMT and CURS concentrations. Notably, as expected for biological systems, high expression of all pathways genes does not necessarily result in optimal production.

The benefit of combinatorial experimentation compared to sequential experimentation is exemplified in Fig. 2C. When the concentration of C3H is low, changing COMT concentration has a limited impact on production. However, this impact increases when the concentration of C3H is high. The relationship between COMT and C3H, also true for other enzymes (Sup. Figure 1), can only be captured through combinatorial optimization and is missed when factors are optimized sequentially.

After the simulation of the full factorial library, DoE fractional factorial designs were simulated selecting experiments according to four different designs (resolution V, resolution IV, resolution III, and PB) or random sampling. These designs differ in the number of strains to build and test. The resolution V design requires the construction of 64 strains and ensures that main effects and two-factor interactions are free of confounding. The resolution IV design requires the construction of 16 strains but two-factor interactions are confounded among each other. Finally, PB and resolution III designs require the construction of 12 and 8 strains respectively but confound main effects with two-factor interactions. In resolution III designs main effects and two-factor interactions are completely confounded and, in PB designs, the correlation between these coefficients is partial. While in random designs any of the strains can be constructed, only a fraction of the strains are selected in DoE fractional designs. This ensures orthogonality in the desired columns and allows a clear estimation of the linear model coefficients (Sup. Figure 2).

### 3.2 Pathway optimization: predictions of optimal strains

Given a set of enzymes (factors) and enzyme concentrations (levels), we analyzed the capacity of different DoE fractional factorial designs to find the enzyme concentration levels that optimize curcumin production. The performance of these designs was compared to random construction of the same number of strains. Each selected set of experiments was used to train a linear model containing main effects and two-factor interactions. Then, significant coefficients were determined by ANOVA. Enzymes with significant main effects are important for production. If an enzyme with a significant effect has a positive coefficient, it should be present at high concentration in the optimal strains. In contrast, an enzyme with a significant negative effect should be present in low concentrations in the optimal strains. For enzymes with insignificant main effects, production should not change regardless of the chosen concentration. Considering these criteria, we computed the frequency at which each strain is predicted as optimal by each design permutation or random sampling. This process was repeated assuming 5% or 20% noise in the data. Predictions were compared to the “ground truth”, defined by the curcumin production of the full factorial library obtained with the kinetic pathway model. We show here the results assuming 20% noise in the production data.

**Fig. 3:**
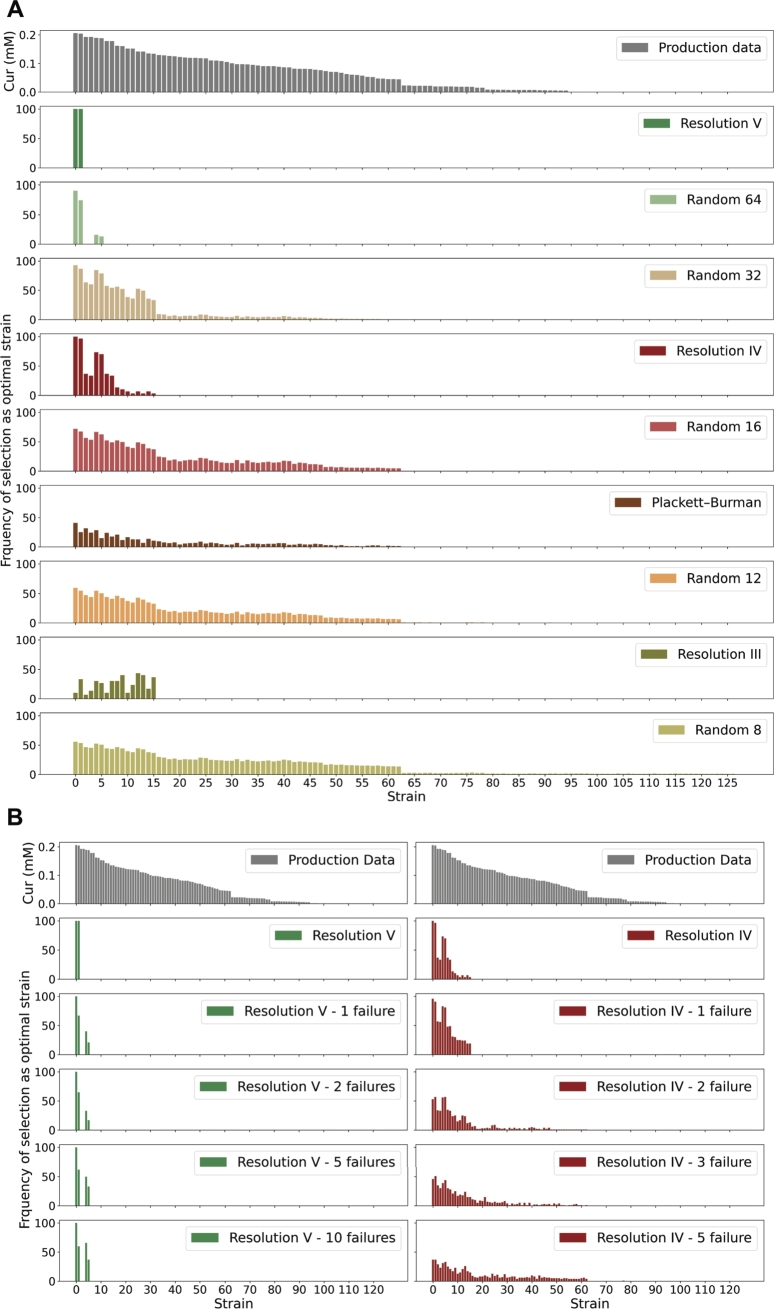
**A.** Prediction of optimal strains based on linear models trained with data from factorial designs and random sampling assuming 20% noise. **B.** Prediction of optimal strains by linear models trained with resolution V and IV factorial designs simulating the inability to construct some of the required strains assuming 20% noise.

The full factorial data shows the presence of two strains with equal performance, characterized by high expression of C3H, CURS, and DCS, low expression of FCS, COMT, and CCOAOMT, and unaffected by the expression level of TAL (Fig. 3A). Only resolution V and IV designs guarantee the identification of both or one of these two optimal strains (Fig. 3A). The resolution V design only suggests two strains as top producers, and the random selection of 64 strains results in the suggestion of four strains. However, the resolution IV design might suggest the construction of up to 16 new strains. Still, the targeted construction of the 16 strains required by the resolution IV design is more efficient than the random construction of 32 strains. When a resolution IV design is used one of the optimal strains is always suggested, and the total number of strains to build and test is reduced. When designs with lower resolution are chosen, the probability of finding the best strains from the full factorial library markedly decreases. Only in the case of the resolution III design, the number of suggested strains to construct is lower than in the random control (Fig. 3A).

Linear models trained with data from fractional factorial designs are usually validated based on their capacity to optimize production [1], [18], [10]. These models need to be trained with data from all the planned experiments in order to avoid losing important information. Therefore, the generation of data for validation would require the construction and testing of additional strains. However, here, the use of *in silico* data allows the validation of the models when predicting the production of all the strains in the full factorial library. Models trained with data from resolution V designs or the random sampling of 64 strains showed the best performance on the full factorial data (R^2^ = 0.83 ± 0.00). They were followed by resolution IV (R^2^ =0.82 ±0.01) and resolution III designs (R^2^ =0.72±0.03). Models trained with PB designs (R^2^ =0.44 ±0.30) or the random sampling of 32 (R^2^ =0.75 ±0.19), 16 (R^2^ =0.49 ±0.16), 12 (R^2^ =0.13 ±1.50), or 8 (R^2^ =-0.35 ±2.54) strains showed lower performance and higher standard deviations (Sup. Figure 3).

For the resolution IV design, we further studied whether including the best two producer strains in the design influenced the prediction of the top strains. In 60% of all the permuted resolution IV designs, the optimal strains were not included, which did not affect the predictions (Sup. Figure 4).

During *in vivo* studies experimental limitations might hinder the construction of some of the required strains for a design. Hence, we studied the robustness of resolution V and IV designs to missing strains. The performance of the resolution V design was minimally affected when up to 10 strains (16%) were excluded from the design. While the number of strains to construct in order to find the optimal production increased from 2 to 4, at least one of the two best producers was always suggested (Fig. 3B). When the resolution IV design was used, excluding one strain from the design (6% of the library) had a minor impact on predictions. However, when 2 (13% of the library) or more strains were omitted, the probability of finding the best strain decreased, and the number of wrongly suggested optimal strains increased (Fig. 3B).

### 3.3 Pathway insights: identification of significant factors and interactions

The coefficients of the linear models are used to predict the optimal strains. Besides, they unveil the effect of each enzyme on curcumin production, improving the understanding of the studied pathway. These insights can then be used to guide following DBTL cycles that focus on the factors with the strongest influence on the response. Moreover, they can point to relevant interactions between factors that enhance the knowledge of the pathway. Here we assess the capacity of each of the DoE fractional factorial designs or random samples to identify significant main effects and interactions. The correct identification of these coefficients explains, in turn, the capacity of each design to find the optimal production strains.

**Fig. 4:**
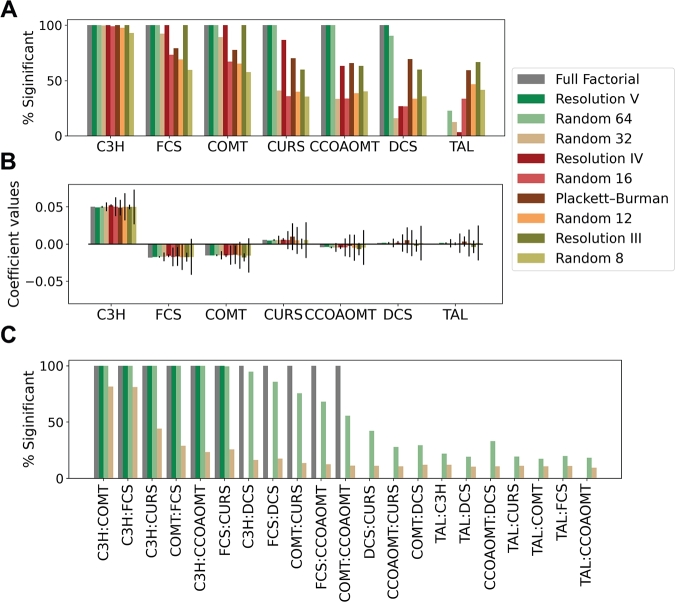
Estimation of linear model coefficients using data from fractional designs and random samples given 20% noise in the response **A.** Frequency of the identification of main effects as significant. **B.** Estimated coefficients for each main effect. Mean coefficients and standard deviations of all possible permutations of the design or random samples are shown. **C.** Frequency of the identification of 2-factor interactions as significant using data from full factorial, resolution V, random 64, and random 32 designs.

The concentration of C3H is the factor with the strongest influence on production. Regardless of the level of noise, all the DoE designs identify C3H as a significant main effect with a positive influence on production (Fig. 4A, B). The importance of this factor is also captured when 64 or 32 strains are randomly sampled. However, when 16, 12, or 8 strains are randomly selected, this effect is missed in 1%, 2%, and 7% of the experiments, respectively (Table 1).

**Table 1:** Frequency (expressed as a percentage) of main effects identified as significant by ANOVA using data from different fractional factorial designs or random strain sampling.

		C3H	FCS	COMT	CURS	CCOAOMT	DCS	TAL
Resolution V	5% noise	100	100	100	100	100	100	0
	20% noise	100	100	100	100	100	100	0
Random 64	5% noise	100	100	100	100	100	90.4	22.6
	20% noise	100	100	100	100	100	90.4	22.6
Random 32	5% noise	100	99.8	99.4	89.2	79.7	56.8	45.2
	20% noise	99.8	92.3	89.3	41.1	33.6	15.9	12.4
Resolution IV	5% noise	100	100	100	100	100	63.3	63.3
	20% noise	100	100	100	88.7	63.3	26.7	3.3
Random 16	5% noise	99.9	95.6	93.5	73.2	79.1	69.4	76.8
	20% noise	98.9	73.4	67.2	36	33.9	26.5	33.8
Plackett-Buramn	5% noise	100	95.7	93.6	93.6	89.8	90.1	87.9
	20% noise	100	79.2	77.6	70.1	65.8	69.4	59.4
Random 12	5% noise	99.7	94	90.2	72.8	77.6	68.9	82.9
	20% noise	97.7	69.1	65.6	40.1	38.6	33.7	46.8
Resolution III	5% noise	100	100	100	93.3	90	86.7	96.7
	20% noise	100	100	100	60	63.3	60	66.7
Random 8	5% noise	98	88.5	83.2	61.2	72.9	65.4	73.7
	20% noise	93	59.7	57.7	35.7	40.4	35.9	41.6

Resolution V, IV, and III designs are always able to identify the negative effect of FCS and COMT. However, 6% to 20% of the PB designs, depending on the level of noise, are unable to capture this behavior (Fig. 4A, B, Table 1). Similarly, the ability to identify the importance of these factors is lost when less than 64 strains are randomly sampled, especially when the level of noise increases.

Given 5% noise, resolution V and IV designs, and the random selection of 64 strains, allow the identification of the positive effect of CURS and the negative effect of COMT (Fig. 4A, B, Table 1). However, when the level of noise increases, the chances of identifying these effects with resolution IV designs decrease to 89% and 63%, respectively. Yet, the likelihood of finding these effects is doubled when compared to the random selection of 32 strains. PB and resolution III designs show similar performance in identifying the importance of these genes. However, these designs are unable to correctly estimate the effect of CURS and COMT on the response as indicated by the high standard deviations of the coefficient values (Fig. 4B). Notably, these designs show better performance than their random counterparts.

DCS and TAL are the factors with the smallest coefficients and, therefore, the smallest impact on production. However, while the expression level of DCS significantly affects production, modifying the expression of TAL does not change curcumin titers. The only design able to capture this behavior is the resolution V design, which outperforms the random selection of 64 strains. Other designs, based on DoE or a random selection of strains, are unable to distinguish the effect of these two genes (Fig. 4, Sup. Table 1).

The inability to correctly identify significant main effects is the reason for the incorrect prediction of optimal strains by the designs (Fig. 3A). For instance, models trained with resolution V designs only predict optimal strains with high concentrations of CURS and DCS, and low concentration of CCOAOMT. However, some of the linear models trained with other designs miss the relevance of these enzymes. They incorrectly suggest strains with low CURS and DCS concentration and/or high CCOAOMT concentration as optimal.

In addition to the determination of main effects, resolution V designs or the random selection of 64 or 32 strains allow the estimation of all the coefficients corresponding to two-factor interactions (Fig. 3C). These interactions point to factors whose effect on the response is affected by the level of another factor. When the full factorial data is used to train a linear model, thirteen significant two-factor interactions are found. All the enzymes but TAL have a significant interaction with C3H and FCS; CURS additionally interacts with COMT, DCS, and CCOAOMT; and COMT and CCOAOMT also show a significant interaction. The presence of a high number of significant interactions highlights the synergistic effect obtained when combining the optimal concentrations of various enzymes and underscores the relevance of combinatorial pathway optimization. However, not all the significant two-factor interactions have the same effect on the response and their absolute coefficients vary from 1.2 ⋅ 10^−2^ to 6.2 ⋅ 10^−4^ (Sup. Figure 5).

Assuming 5% noise in the response, linear models trained with resolution V designs correctly identify the eleven most important interactions, including interactions with absolute coefficients of 10^−3^. When the level of noise increases to 20% this design still allows the identification of the six most important two-factor interactions, with absolute coefficients above 2.8 ⋅ 10^−2^. Regardless of the level of noise, models trained with resolution V designs prevent the incorrect identification of insignificant interactions (false positives), frequently found when randomly selected strains are used for model training (Fig. 3C).

**Fig. 5:**
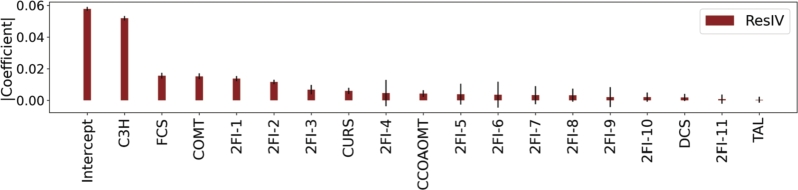
Absolute values of model coefficients trained with data from permutations of a resolution IV design. The mean coefficient and standard deviation of main effects and confounded two-factor interactions (2FI) are shown.

When resolution IV designs are used to train linear models, specific two-factor interactions cannot be determined. However, the estimated coefficients of the confounded interactions give information on their relative importance compared to the main effects. Fig. 5 shows how two-factor interactions 1 and 2 have an effect on the response similar to the main effect of COMT. Likewise, the effect of two-factor interaction 3 is similar to the main effect of CURS. Therefore, these designs are able to clearly identify that the effect of interactions in the studied system should not be ignored. Notably, resolution IV designs with up to three missing strains are also able to correctly estimate the relevance of the two-factor interactions (Sup. Figure 6). Considering this, the best strains in the design space could be found using a sequential experimentation approach. For instance, a resolution IV design could be first used to identify C3H, FCS, and COMT as the most important main effects, relative to the importance of two-factor interactions. In a second DBTL round, the expression level of these genes could be fixed according to the sign of their coefficients. Then, a resolution V design with the remaining 4 factors could be performed. In this case, the resolution V design involves the construction of 16 strains and is equivalent to a full factorial design. This approach ensures the identification of the optimal strains with a total of 32 experiments.

Finally, the partial correlation between main effects and interactions in PB designs should allow the identification of some interactions. Using subset regression the factors and interactions that result in models with better fit could be identified. However, subset models only consistently predicted the importance of C3H and failed to find significant interactions. These results together with the low R^2^ of models trained with PB designs (Sup. Figure 3) showcase the inappropriateness of this design for pathway understanding and optimization.

### 3.4 Comparison of linear models and random forest

Machine learning (ML) algorithms can be used as an alternative to linear models to gather the information obtained during experimentation based on DoE designs [19]. Although recovering information from these models is harder than from linear models, ML algorithms can recognize more complex patterns within the data. As an example of a ML algorithm, we tested the ability of random forest models trained using 10-fold cross-validation to predict the best-performing strains given data from random samples or DoE designs. However, training these models with 32 or fewer experiments often resulted in negative R^2^ values for some of the iterations. Even when only models with R^2^ coefficients above 0.6 were used, randomly selecting experiments or using DoE factorial designs resulted in equally bad predictions of best strains (Sup. Figure 7). These results did not change when all the strains from a design were used for model training. This strategy, equivalent to the training of linear models, did not include cross-validation and avoided losing information due to a reduced number of strains during training. Hyperparameter tuning did not improve the performance of the random forest models. Therefore, given the small number of experiments required when considering the optimization of seven factors, linear models outperform random forest. However, the ability of ML models to benefit from training data based on DoE designs if the number of factors and, therefore, experiments increases, remains unexplored.

## 4 Discussion

DoE involves the design of experiments using fractional factorial designs and their analysis using linear models and ANOVA. Although there are numerous tools to perform and analyze these designs [7], [8], [9], clear recommendations on which designs to use for pathway optimization are missing. Here we showed how fractional factorial designs can be used to find the optimal concentration of a pathway's enzymes and understand the impact of factors on production. In both cases, the resolution V design, which requires half of the experiments of a full factorial, excels. It provides the same information as the full factorial design and finds the strains with the best curcumin titers. When this design is used, all main effects are correctly identified as well as the most important two-factor interactions (Fig. 4). We highlight the relevance of these interactions to understand and improve production, as designs where main effects and interactions are confounded struggle to find the optimal strains (Fig. 3A). However, the identification of a significant interaction does not necessarily reflect a biological mechanism. For instance, factors representing all enzymes but TAL are included in significant interactions with C3H. This is due to the requirement of high C3H concentration to obtain high levels of curcumin. It does not mean that all these enzymes physically interact with C3H but it could lead to hypotheses aiming to explain the importance of this enzyme for the pathway functioning.

We propose resolution IV designs as the best trade-off between information gain and experimental effort, and the best option to initially screen the effect of factors in the response. The key strength of this design is the lack of confounding among main effects and interactions, which allows the confident identification of main effects. Besides, although two-factor interactions are confounded, these designs allow weighting their importance compared to the individual effects. Moreover, these designs provide a solid knowledge basis of the system under study that can be expanded in different directions depending on the experimental goal. Here, we show how, when the aim is to find the best possible production given the initial factors and levels, the most important main effects can be fixed. Then a resolution V design can be performed on the remaining factors. In this case, two-factor interaction coefficients involving the most important (fixed) factors will not be estimated, but the optimal strain will be found. Alternatively, if the goal is to find these coefficients, original resolution IV designs could be augmented using D-optimal designs. These designs select experiments from the full factorial that allow the clarification of the desired interactions by minimizing the variance of the model coefficients [6]. Finally, when the researcher aims at expanding the original design space, the number of levels of the most relevant factors could increase following the direction indicated by the linear model coefficients. Alternative designs such as Box-Behnken designs that include three levels per factor could be used to train response surface models [10], [6], in this case, testing higher concentrations of C3H and lower concentrations of FCS and COMT.

In this study, the use of a kinetic model allowed the simulation of a full factorial design and the comparison of fractional designs without a limitation on throughput (*i.e.* number of strains to test and build). This comparison was performed considering realistic scenarios including noise and datasets with missing information due to, for instance, problems during strain construction. However, during the *in vivo* optimization of pathways, the achievable throughput is a critical parameter that should determine how the optimization process is performed. Given the throughput, we recommend fixing the number of factors to screen to be able to obtain resolution IV designs. The advent of biofoundries that automate the strain construction process is continuously increasing the capacity to build strains [20], [21], [22]. This increase should be accompanied by high-throughput, automated cultivation and screening protocols as well as automated data collection [23]. Scaling these processes will allow the assessment of numerous factors in screening studies that should go beyond pathway engineering to include optimization at the metabolic and bio-process levels.

Scripts for the generation and analysis of the designed libraries, the prediction of optimal strains and the analysis of the desigs using ML, as well as additional data are available at Gitlab.

## Declaration of generative AI and AI-assisted technologies in the writing process

During the preparation of this work the authors used ChatGPT 3.5 in order to improve the readability of the text. After using this tool, the authors reviewed and edited the content as needed and take full responsibility for the content of the publication.

## CRediT authorship contribution statement

**Sara Moreno-Paz:** Conceptualization, Data curation, Formal analysis, Investigation, Methodology, Software, Validation, Visualization, Writing – original draft. **Joep Schmitz:** Conceptualization, Supervision, Writing – review & editing. **Maria Suarez-Diez:** Conceptualization, Funding acquisition, Supervision, Writing – review & editing.

## Declaration of Competing Interest

Joep Schmitz (JS) is employed by DSM-firmenich.

## Data Availability

Scripts for the generation and analysis of the designed libraries, the prediction of optimal strains and the analysis of the desigs using ML, as well as additional data are available at Gitlab.

## Appendix A Supplementary material

MMC Supplementary Figures. MMC
