# Supplementary material for: In silico analysis of design of experiment methods for metabolic pathway optimization
Source: Comput Struct Biotechnol J. 2024 May 3;23:1959–67. doi: 10.1016/j.csbj.2024.04.062 (PMC11087228; doi:10.1016/j.csbj.2024.04.062)
Supplement: MMC — Supplementary Figures. [file mmc1.pdf]

# 7 Supplementary Figures

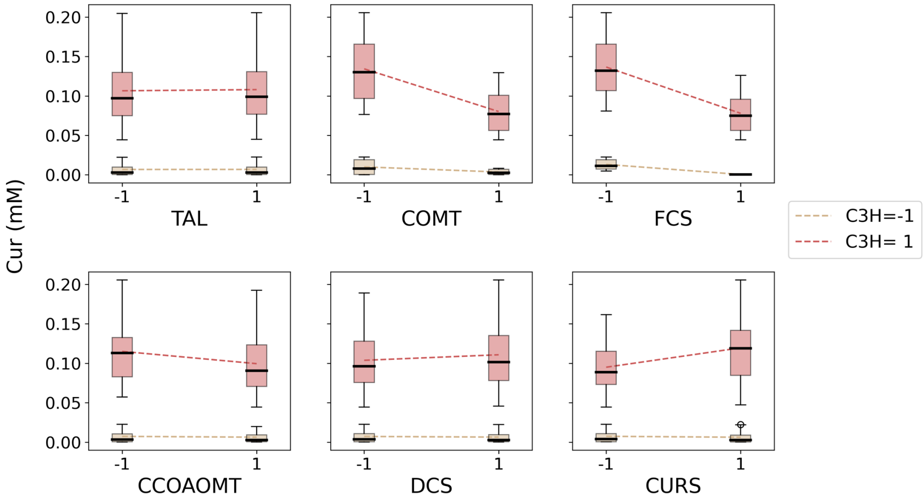


Sup. Figure 1: Sequential vs. combinatorial experimentation. Example of how the concentration of the C3H enzyme affects the impact of changing other enzyme concentrations on curcumin (Cur) production. -1, low enzyme concentration; 1, high enzyme concentration.

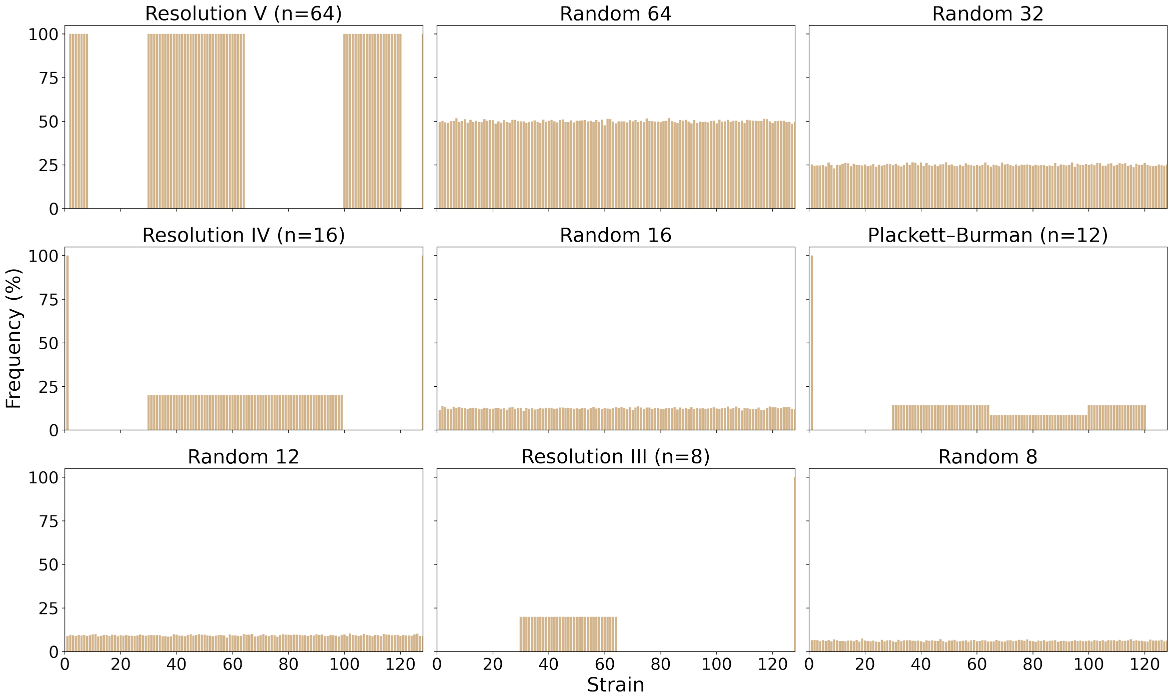


Sup. Figure 2: Frequency of strain selection by the different design approaches including factorial designs and random sampling.

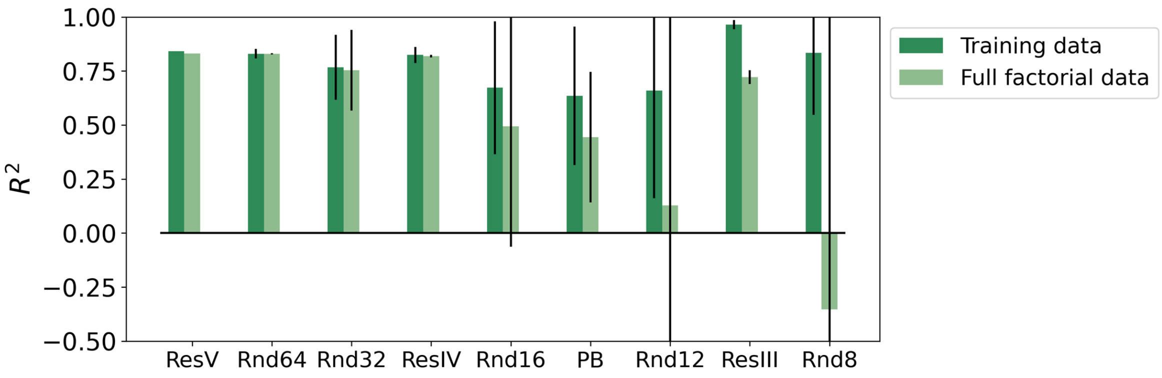


| | Training data | Full factorial data |
|---|---|---|
| **Resolution V (ResV)** | 0.84 ± 0.00 | 0.83 ± 0.00 |
| **Random 64 (Rnd64)** | 0.83 ± 0.02 | 0.83 ± 0.00 |
| **Random 32 (Rnd32)** | 0.77 ± 0.15 | 0.75 ± 0.19 |
| **Resolution IV (ResIV)** | 0.82 ± 0.04 | 0.82 ± 0.01 |
| **Random 16 (Rnd16)** | 0.67 ± 0.31 | 0.49 ± 0.56 |
| **Plackett Burman (PB)** | 0.63 ± 0.32 | 0.44 ± 0.30 |
| **Random 12 (Rnd12)** | 0.66 ± 0.50 | 0.13 ± 1.50 |
| **Resolution III (ResIII)** | 0.96 ± 0.02 | 0.72 ± 0.03 |
| **Random 8 (Rnd8)** | 0.83 ± 0.29 | -0.35 ± 2.54 |

Sup. Figure 3: Coefficient of determination ($R^2$) of linear models trained with fractional factorial designs or random strains. Performance when predicting the training data and the data from the full factorial library is shown. The mean $R^2$ and its standard deviation considering all possible permutations of the design or random samples are shown.

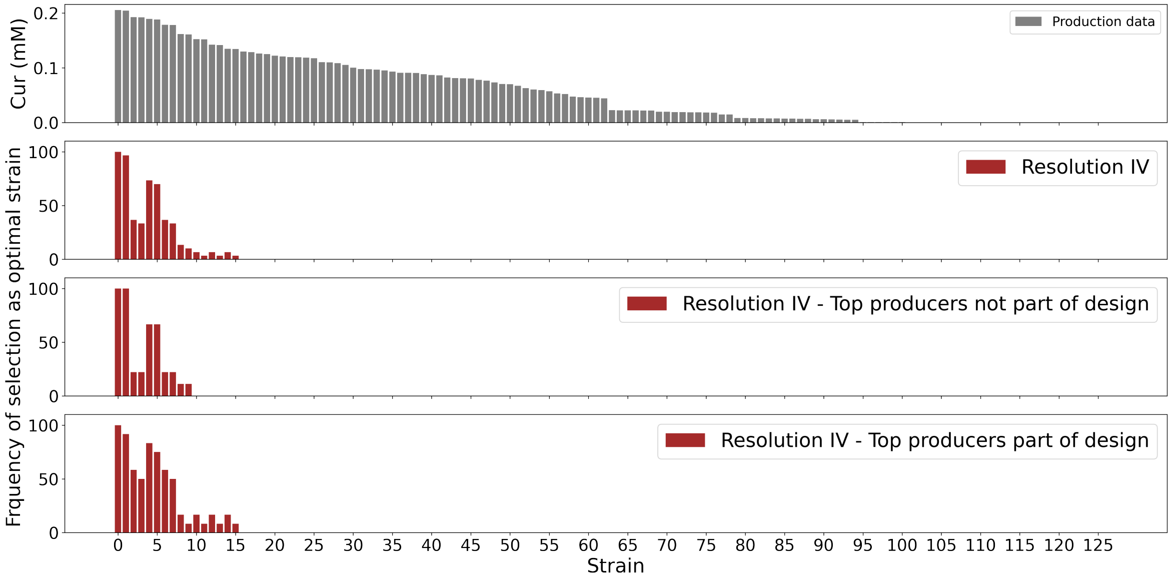


Sup. Figure 4: Prediction of best strains by linear models trained with all resolution IV designs, resolution IV designs that exclude the two best strains in the design (60% of the designs) or that include them (40% of the designs).

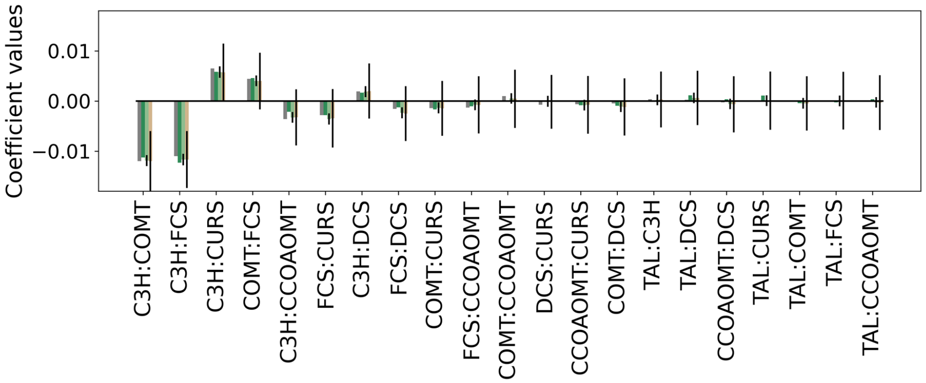


Sup. Figure 5: Estimated coefficients for each two-factor interaction using data from resolution V, random 64 and random 32 designs. 20% noise in the response is used. The mean coefficient and standard deviation of the coefficients considering all possible permutations of the design or random samples are shown.

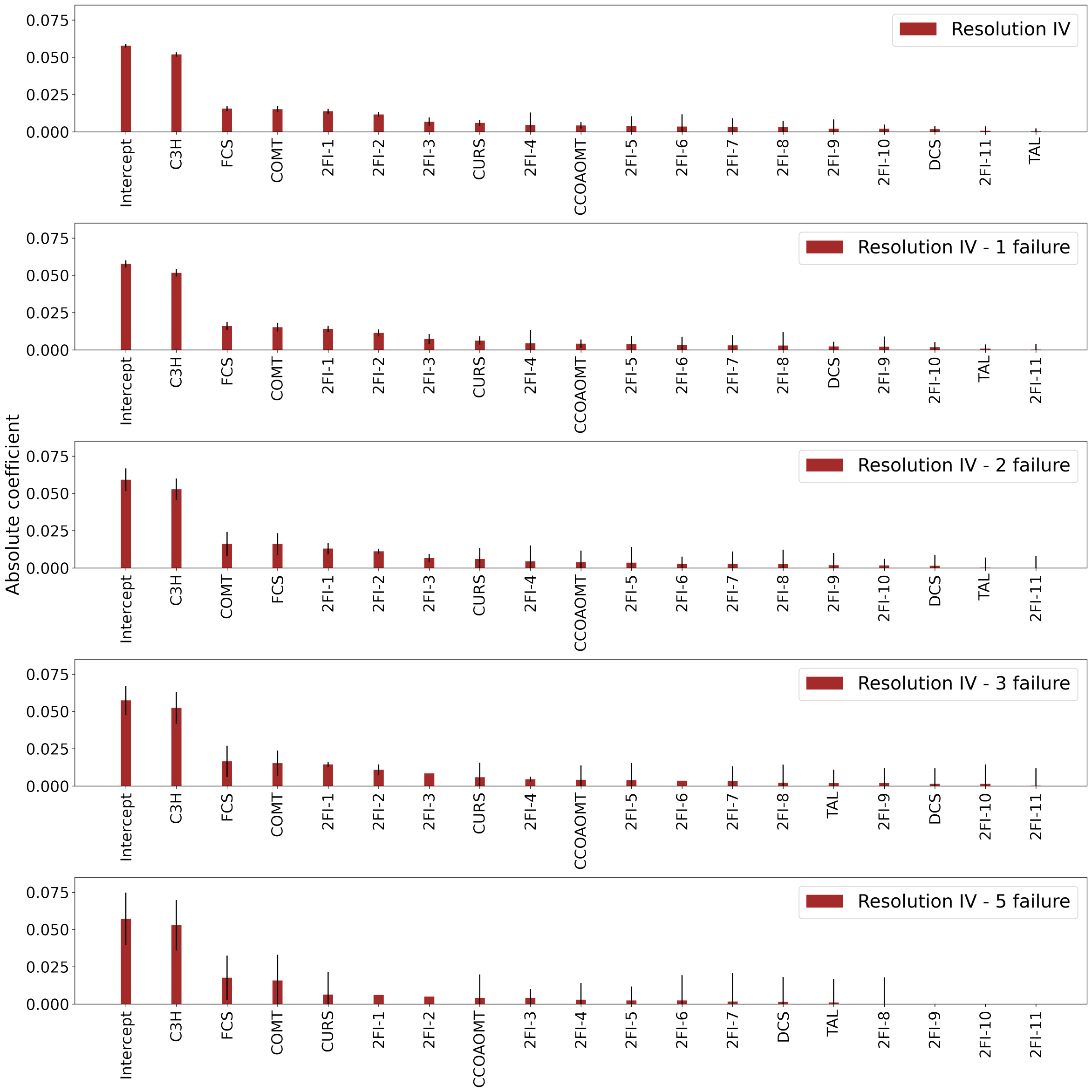


Sup. Figure 6: Absolute coefficients of the main effects and the 11 confounded two-factor interactions (2FI) estimated using data from resolution IV designs with missing strains. The mean coefficient and standard deviation of the coefficients considering all possible permutations of the design are shown.

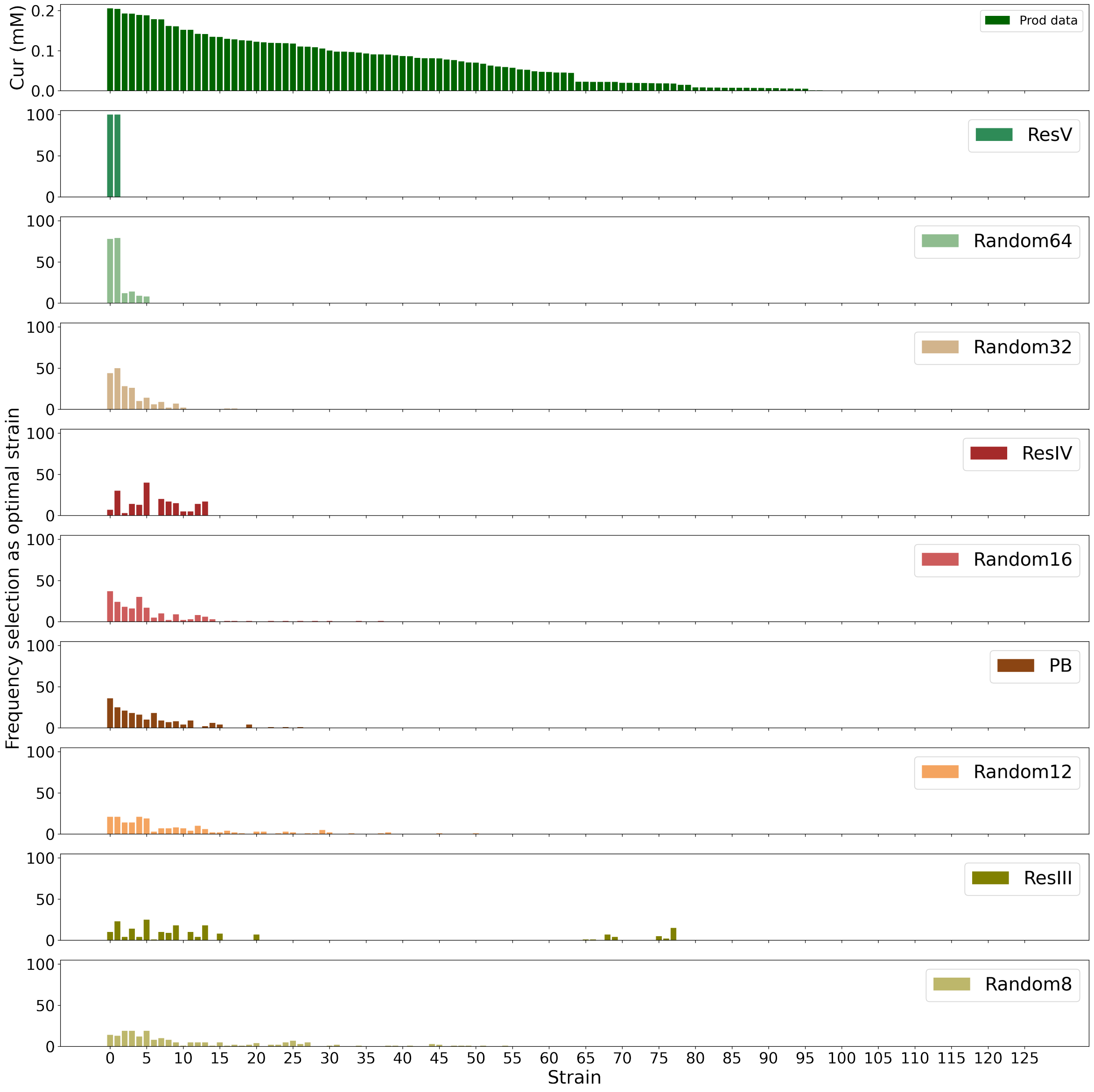


Sup. Figure 7: Prediction of best strains by random forest models trained with data from different factorial designs or random selection of strains assuming 20% noise in the response.
